# Ozonation of Decalin as a Model Saturated Cyclic Molecule: A Spectroscopic Study

**DOI:** 10.3390/molecules26185565

**Published:** 2021-09-13

**Authors:** Boris G. Ershov, Nadezhda M. Panich, Gennadii L. Bykov, Alexander L. Kustov, Vladimir G. Krasovskiy, Leonid M. Kustov

**Affiliations:** 1 A.N. Frumkin Institute of Physical Chemistry and Electrochemistry RAS, 31 Leninsky Prospect, bldg. 4, 119071 Moscow, Russia; ershov@ipc.rssi.ru (B.G.E.); panich@inbox.ru (N.M.P.); bykov@ipc.rssi.ru (G.L.B.); 2 N.D. Zelinsky Institute of Organic Chemistry RAS, 47 Leninsky prosp., 119991 Moscow, Russia; kyst@list.ru (A.L.K.); miyusha@mail.ru (V.G.K.); 3 Institute of Ecotechnologies and Engineering, National University of Science and Technology MISiS, 4 Leninsky prosp., 119049 Moscow, Russia; 4 Chemistry Department, Moscow State University, 1 Leninskie Gory, bldg. 3, 119991 Moscow, Russia

**Keywords:** ozonolysis, decalin, carboxylic acids, decomposition

## Abstract

Ozonolysis is used for oxidation of a model cyclic molecule-decalin, which may be considered as an analog of saturated cyclic molecules present in heavy oil. The conversion of decalin exceeds 50% with the highest yield of formation of acids about 15–17%. Carboxylic acids, ketones/aldehydes, and alcohols are produced as intermediate products. The methods of UV-visible, transmission IR, attenuated total reflection IR-spectroscopy, NMR and mass-spectrometry were used to identify reaction products and unravel a possible reaction mechanism. The key stage of the process is undoubtedly the activation of the first C-H bond and the formation of peroxide radicals.

## 1. Introduction

Ozone is known to be an efficient oxidizing agent and is widely used in organic syntheses and in purification of water by the complete oxidation of traces of organic compounds present in water. Ozone is characterized by high reactivity with unsaturated bonds, aromatic structures, and fragments containing electron-donor atoms and groups [1,2,3,4]. Thus, the use of ozone and ozonation in some cases provides intensification of chemical processes.

Ozonation is an alternative to other known methods of synthesis of oxygenated organic compounds in the oxidation of cycloalkanes. However, ozonation of naphthenic molecules has been studied to a much lesser extent than reactions with unsaturated and aromatic substrates. This can be understood considering similarities in the properties of alkanes and cycloalkanes, which are quite inert toward oxidizing agents, except for C3 and C4 cycles, which are much like olefins.

Ozonation of cyclic saturated hydrocarbons results in a high yield of tertiary alcohols (from 76 to 100%) while maintaining the primary configuration [5,6].

Noncatalytic ozonization of adamantane on silica gel has been shown to result in the formation of adamantanol at −78 °C [7]. The use of ozone as an oxidizing agent in the selective oxidation of alkanes (noncyclic and saturated molecules) has been reported in combination with metal complexes as catalysts, for instance, iron porphyrins [8] or manganese polyoxometalates [9,10]. There are also examples of ozonation of ethers with the formation of esters [11], whereas amines produce amides and N-dealkylation products when reacting with ozone [12,13,14].

The formation of cyclodecanone was observed in the course of ozonation of a suspension of cyclodecane in water in an acidic medium [15]. The main product was cyclodecanone with a minor amount of cyclodecanol (1 h, 25 °C). Carboxylic acids (10-oxodecanoic acid and decanedioic acid) was also found in equimolar amounts in addition to cyclodecanone. The use of a neutral pH value was detrimental for the selectivity of cyclodecanone.

Ozonation of decalins [16] proceeds via the activation of the secondary carbon atoms by converting decalin into carbonyl compounds. Because of poor solubility in water, the ozonation of cis-decalin was performed in nitromethane at 5 °C over 30 min, and complete conversion was found with the formation of cis-9-hydroxydecalin and a small amount of the trans-isomer. The conversions and yields of cis-9-hydroxydecalin were as high as 99% and 57%, respectively, with the corresponding ketone being the second major product. The ozonation of trans-decalin under the same conditions resulted in much lower conversions, with trans-9-hydroxydecalin being the main product.

A concerted mechanism of hydrogen transfer as the first step in the hydroxylation process was proposed [17]. Noteworthy is that only H_2_O_2_ was formed as a byproduct in these processes, which makes this approach environmentally attractive.

Unsaturated organic compounds, including alkenes and aromatic molecules, are vigorously oxidized by ozone. Alkanes, on the other hand, exhibit a high resistance to oxidation, and this resistance increases with decreasing temperature. Moreover, in isooctane, hexane, and heptane at 201 K, ozone does not decay, and its content significantly exceeds 0.1 mol/L [18].

It can be assumed that the presence of a mobile hydrogen atom at the tertiary carbon atom significantly increases the ability of decalin to be oxidized by ozone. This should contribute to understanding the role of the spatial structure of saturated cyclic hydrocarbons in oxidation.

Thus, the ozonation of decalin is definitely underexplored, and the goal of the present study was to perform such research using UV-visible, transmission IR and attenuated total reflection IR spectroscopy as analytical methods to control the process of oxidation. Other spectroscopic techniques (mass-spectrometry and NMR spectroscopy) were also used to understand the nature of the products resulting from ozonation of decalin and to assess its conversion as a model for saturated cyclic molecules present in heavy oil. For instance, ozonation of asphaltenes, including asphaltenes produced after preliminary hydrogenation of the native asphaltenes extracted from heavy oil residues, may be considered as a practically viable method of asphaltene conversion into added-value products. Asphaltenes represent a complicated mixture of hydrocarbons, some of which also contain N and S-heterocycles, but the detailed analysis of this mixture is almost impossible. Simple model molecules such as decalin are close in structure to the components of hydrogenated asphaltenes; therefore, decalin was chosen as a model compound in this study.

## 2. Results and Discussion

### 2.1. Optical Spectra

It was found that the contact of ozone with decalin does not lead to the appearance of a well-resolved characteristic ozone absorption band in an optical spectrum with a maximum at 258 nm, although some residual absorption in the region of 260 nm was seen. Thus, ozone effectively interacts with decalin when passing the ozone-oxygen mixture through decalin, unlike alkanes that do not interact with ozone under the same conditions.

**Figure 1:**
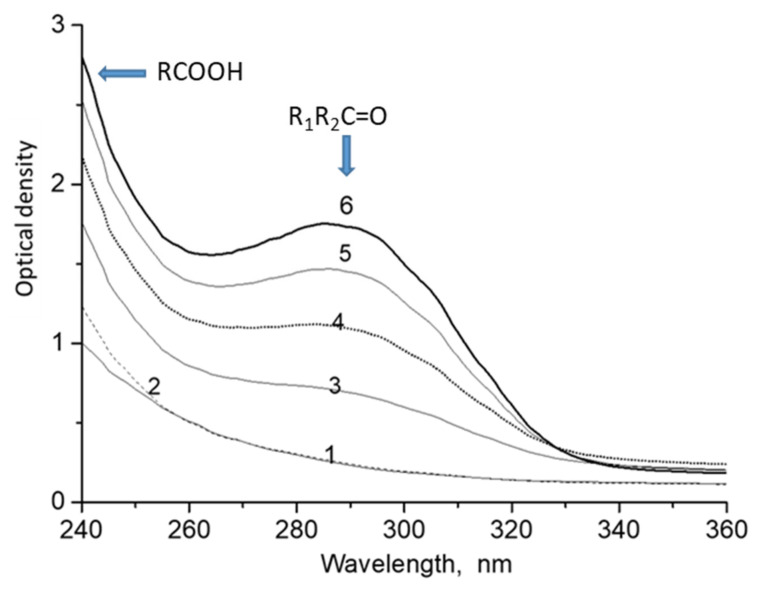
UV-spectra of decalin after ozonation. The optical path, 1 cm. [O_3_] = 100 mg/L. The flow rate of the ozone-oxygen mixture was 2 mL/s. T = 20 °C. Decalin ozonation time (min): 1—0; 2—2; 3—5; 4—10; 5—15 and 6—20.

Decalin ozonation at room temperature was accompanied by the appearance of optical absorption in the UV range (Figure 1). It is assumed that the absorption band at 288 nm is caused by C=O groups and the band at λ ≤ 250 nm by COOH groups [19]. Therefore, we conclude that aldehydes, ketones, and carboxylic acids are formed in addition to hydroxydecalin isomers (E- and Z-decalol isomers). Some of these were identified by mass spectrometry (Supplementary Materials).

The absorption band at 288 nm is attributed to the n→π * transition that is characteristic of aldehydes and ketones. This transition has the lowest energy of all types of electronic transitions. The absorption bands of n→π * transitions are usually observed in the region of λ_max_ > 250 nm. Absorption bands for saturated aldehydes and ketones are usually observed in the region of 280 nm, and the molar extinction coefficient ranges within 13–20 L/cm mol. For instance, the corresponding value for acetone is 13.0, for 2-butanone 17.8, and for 3-pentanone 20.4 [20]. We assume that the carbonyl compounds formed in the course of decalin ozonation are characterized by close values of the molar extinction coefficient; therefore, we used an average value of 17 L/cm·mol. This makes it possible to estimate the concentration of compounds with aldehyde-ketone groups. According to Figure 1, assuming ε = 17 L/cm·mol, the concentrations can be calculated as approximately 2.6 × 10^−2^, 5.0 × 10^−2^, 7.3 × 10^−2^ and 9.2 × 10^−2^ mol/L for the ozonation times of 5, 10, 15, and 20 min, respectively.

The n→π* bands of C=O groups in carboxylic acids and their derivatives have different characteristics compared to ketones and aldehydes: λ_max_ = 200–205 nm and ε = 50–70. The π →π* bands of C=O groups of acids are located in the region of about 200 nm and are characterized by large ε values of 1000–10,000.

**Figure 2:**
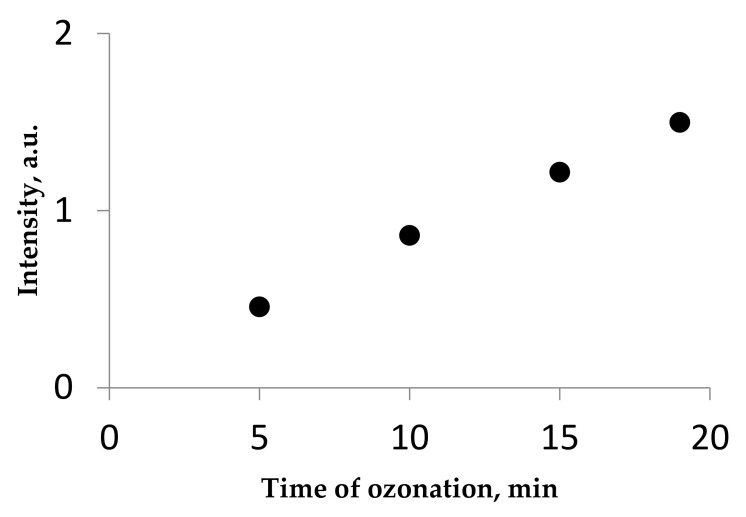
Dependence of the intensity of the UV absorption band at ~288 nm vs. time upon ozonation of decalin. T = 22 °C. [O_3_] = 100 mg/L. Optical path, 1 cm.

A gradual increase of the intensity of absorption bands attributed to the products of ozonation is shown in Figure 1. Figure 2 shows that this dependence is close to the linear function.

**Figure 3:**
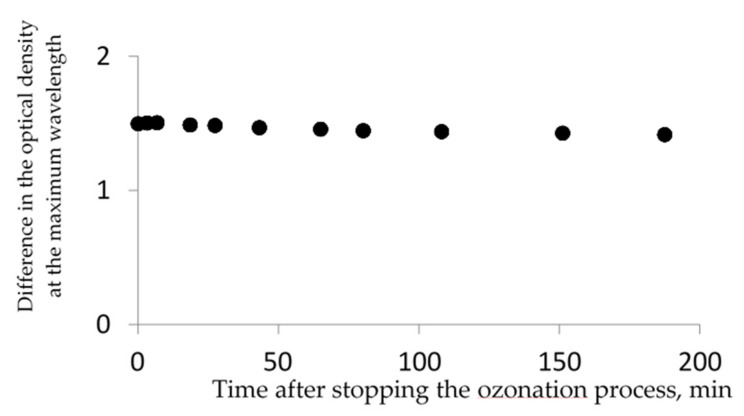
Dependence of the intensity of the absorption band at λ_max_~ 288 nm, i.e., the difference in the optical density at the maximum wavelength vs. time after stopping the ozone flow. Ozonation time 19 min. [O_3_] = 100 mg/L.

The linearity of this dependence can be explained as follows: the decalin oxidation prevails over the further oxidation of the oxygenated products formed, which is quite unusual because the oxidized products are expected to be more reactive than the starting saturated molecule, which is somewhat similar to alkanes. Thus, the products of decalin oxidation are quite stable with the action of ozone. This is also confirmed by the constant value of the absorption band intensity, which remains unchanged upon the completion of the ozonation process (Figure 3).

**Figure 4:**
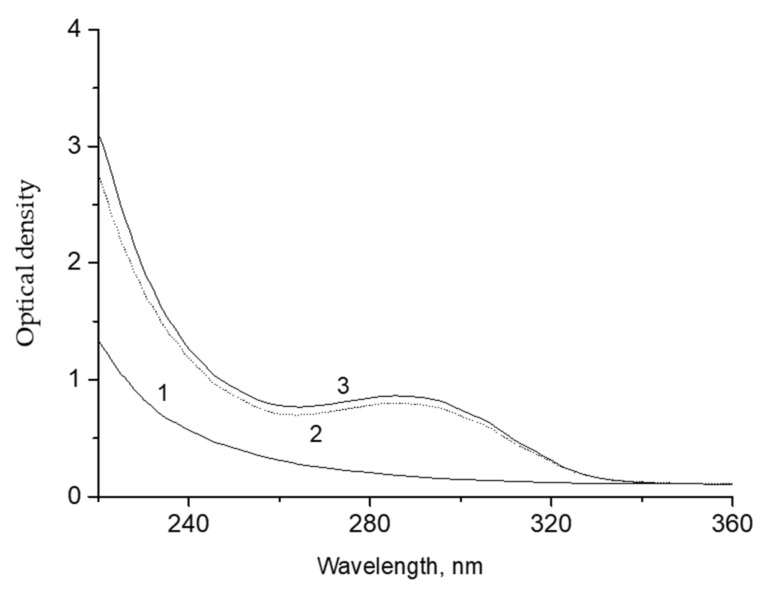
UV spectra of the initial decalin (**1**), decalin phase after extraction with water (**2**), and decalin phase after ozonation for 19 min (**3**). Optical path, 5 mm.

The products of decalin ozonation were extracted into water (the decalin: water ratio was 1:1). The portion of the extracted product, as estimated from the difference in the intensity of the UV-spectrum before and after extraction (Figure 4) did not exceed 5–10%.

**Figure 5:**
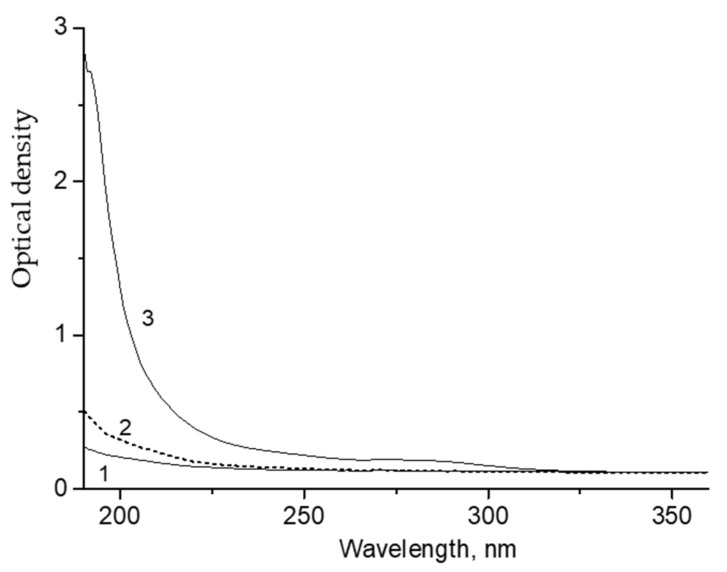
UV spectra of water (**1**), aqueous phase after extraction of initial decalin with water (**2**) and aqueous phase after extraction of decalin ozonated for 17 min (**3**). Optical path, 2 mm.

Absorption spectra of the water extract from the ozonated decalin showed the presence of a band at 288 nm and strongly increasing absorption in the UV region with λ_max_ ≥ 200 nm (Figure 5). It can be concluded that soluble fractions of compounds with C=O groups, and with λ ≤ 250 nm belonging to COOH groups, were extracted into the aqueous medium, i.e., aldehyde, ketone, and carboxylic compounds.

The conversion of decalin during the 70 min experiments determined from the intensities of the spectra was close to 50%, with the highest yield of acids about 15–17%.

The water extracts had a low pH value and the acidity increased with increasing time of ozonation. Thus, pH values of the water extracts were equal to 3.3 and 2.4 after extraction of decalin ozonated for 17 and 70 min, respectively. Therefore, we conclude that carboxylic acids are formed predominantly by ozonation and are transferred to the water phase from the organic solution.

### 2.2. IR Spectra

The transmission IR spectra were first measured after ozonation of decalin for 70 min (Figure S1). A new absorption band at 1717 cm^−1^ appeared in the IR spectra. This band can be assigned to C=O groups in carboxylic acids. A broad band at 3450 cm^−1^ was also observed, which corresponds to the hydrogen-bonded COOH groups of the carboxylic acids formed upon ozonation of decalin.

As the transmission IR spectra demonstrated significant noise, further IR studies were performed using the attenuated total reflection mode (ATR). The assignment of the absorption bands in the IR spectra of cis- and trans-decalins, initial decalin (equilibrium mixture of cis- and trans-isomers) and decalin after ozonation for 15 and 70 min, are presented in Table 1. The comparison of the IR spectroscopic features of the initial mixture of decalin isomers and the mixture treated with ozone for 15 and 70 min, with those of the individual cis and trans-decalin isomers, are presented in Table S1 (Supporting Information).

**Figure 6:**
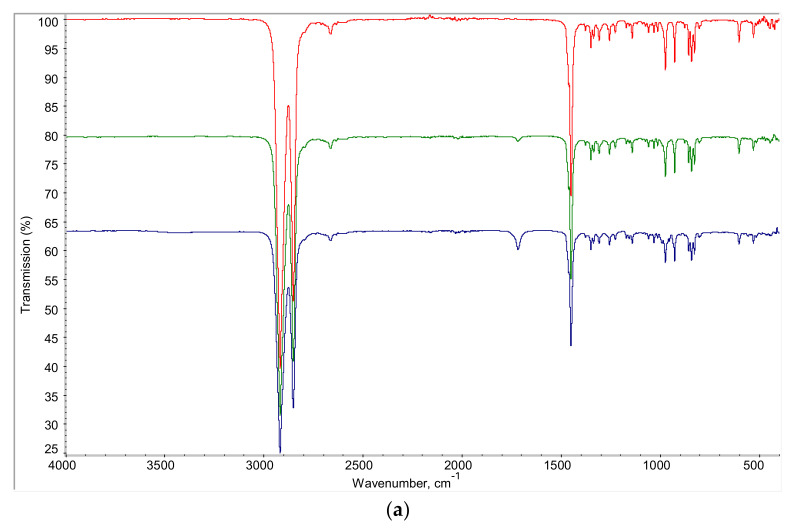

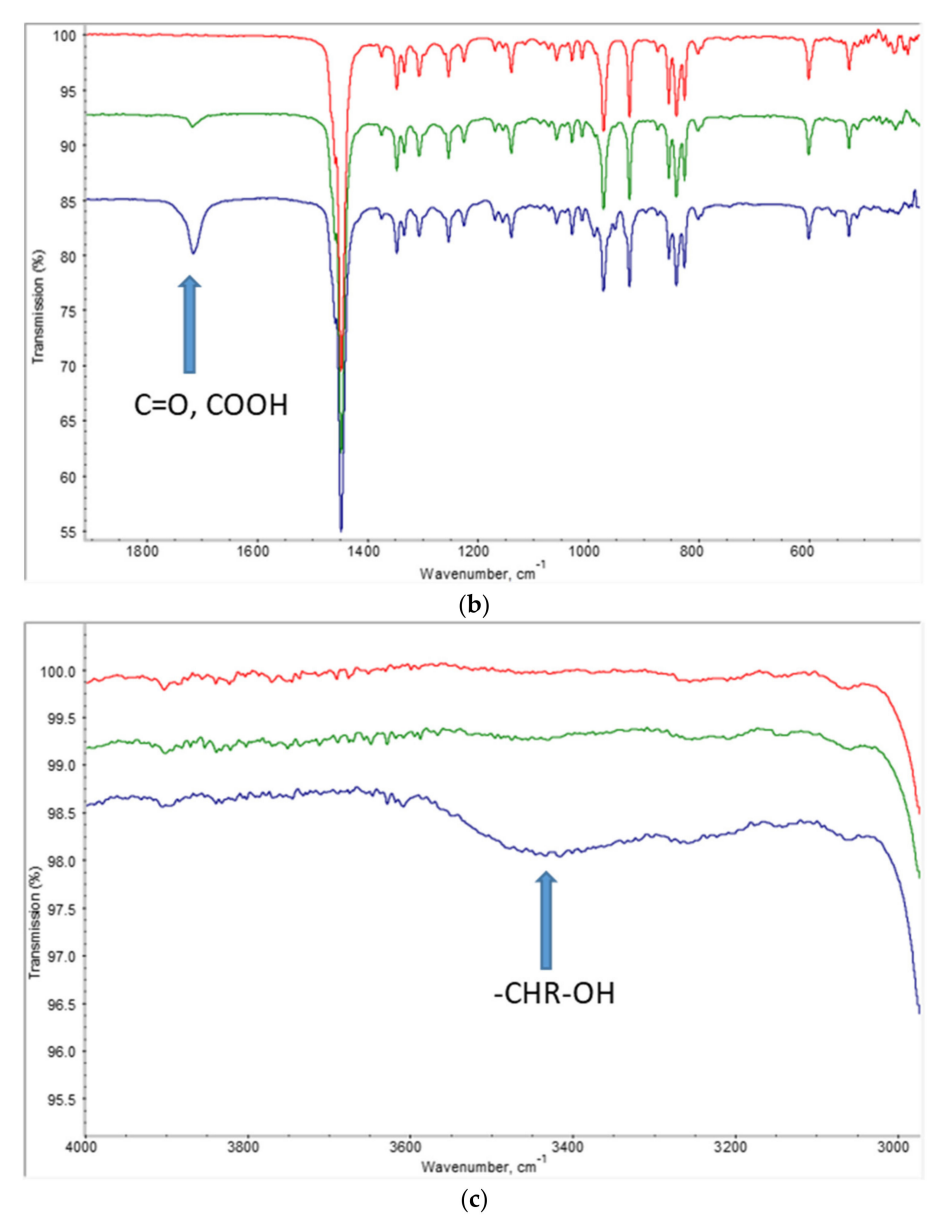
IR-ATR spectra of the initial mixture of cis and trans-decalins (red), and the same mixture after ozonation for 15 min (green) and 70 min (blue): (**a**) survey spectra in the entire frequency range; (**b**) spectra in the region of 2000–400 cm^−1^; (**c**) spectra in the region of 4000–2900 cm^−1^.

The analysis of the measured IR-ATR (attenuated total reflectance) spectra (Figure 6) of the samples of decalin before and after oxidation with ozone for 15 and 70 min, and comparison with the IR-ATR spectrum of the initial decalin (mixture of cis- and trans-isomers) and standard IR spectra of cis-decalin and trans-decalin, showed that the infrared spectrum changed after treatment of decalin with ozone, with new absorption bands appearing in the regions of 3600–3000 cm^−1^, 1720–1710 cm^−1^ and 990–940 cm^−1^ (as well as at 552 cm^−1^). The absorption bands in these regions are characteristic of vibrations of oxygen-containing bonds, namely carboxyl groups, which indicates decalin oxidation by ozone.

The absorption band at 1717 cm^−1^ was an unsymmetric peak, and probably the sum of several absorption bands (Figure 6b) that characterize the vibrations of C=O bonds of carbonyl and carboxyl groups. The process of oxidation of decalin with ozone takes place with the formation of a set of products including alcohols, aldehydes and acids. At the first stage, mainly aldehydes and alcohols are formed, as evidenced by the appearance in the IR spectrum of the reaction mixture after 15 min of ozonolysis of a weak absorption band at 3450 cm^−1^ in the region of stretching vibrations of OH-groups, and a weak absorption band at 1717 cm^−1^ assigned to vibrations of C=O bonds (Figure 6b). The asymmetry of the absorption band of C=O groups suggests the presence of carboxylic groups in the structure of the reaction products at the initial stage of the reaction.

An increase in the ozonolysis reaction time to 70 min led to a significant increase in the intensity of the absorption bands at 3450 cm^−1^ and 1717 cm^−1^. At the same time, the absorption band of the stretching vibrations of OH groups was widened towards 2900 cm^−1^ (Figure 6c) and the asymmetry of the absorption band of vibrations of C=O bonds was preserved (Figure 6b). The broadening of the bands of hydroxyl groups was associated with an increase in their acidity, i.e., along with the alcohol OH groups, the reaction mixture contained more COOH groups, which was also confirmed by an increase in the intensity of the bands in the region of 990–940 cm^−1^ attributed to symmetrical stretching vibrations of C-O bonds (Figure 6b).

Thus, as a result of ozonation, decalin was oxidized with a break of the cycle and the appearance of alcohol, aldehyde, and acid carboxylic groups in the structure of products. An increase in the reaction time increased the content of all three oxygen-containing groups in the reaction mixture in the IR spectrum after 70 min of the reaction, along with a broadening of the absorption band in the region of stretching vibrations of OH groups. A band at 3450 cm^−1^ appeared, related to vibrations of alcohol and carboxylic groups, and an absorption band at 1717 cm^−1^, due to vibrations of C=O bonds. As the intensity increased, this band retained its asymmetry.

The IR spectrum of ozonated decalin measured after 4 days did not change compared with the fresh ozonated sample. Thus, the compounds formed in the course of decalin ozonation are quite stable and do not decompose while being in contact with air.

The intensities of the bands at 1717 cm^−1^ assigned to C=O groups, and at 3450 cm^−1^ assigned to COOH groups of the carboxylic acids increased with duration of ozonation (Figure 6c). This means that the amount of oxidation products accumulated in the system increased with time of ozonation in accordance with optical data.

After extraction with water, the intensities of the products of decalin ozonation decreased insignificantly. Figure S2 shows the IR spectra of ozonated decalin before and after extraction with water. It is clearly seen that the intensity of the carbonyl band decreased by 5–8%. These data are in full agreement with the UV-spectroscopic data.

### 2.3. NMR Spectra

**Figure 7:**
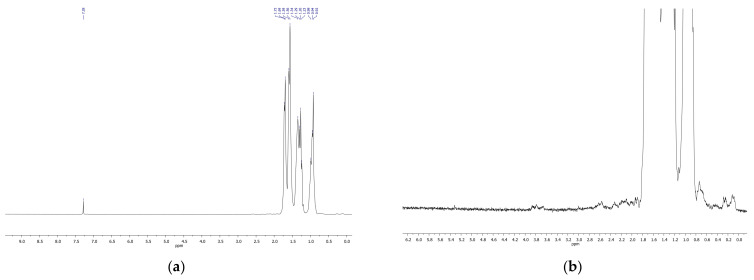
^1^H-NMR spectrum of the initial mixture of cis- and trans-decalins in a wide range of chemical shifts (**a**) and in the narrow range of 0–6 ppm (**b**).

**Figure 8:**
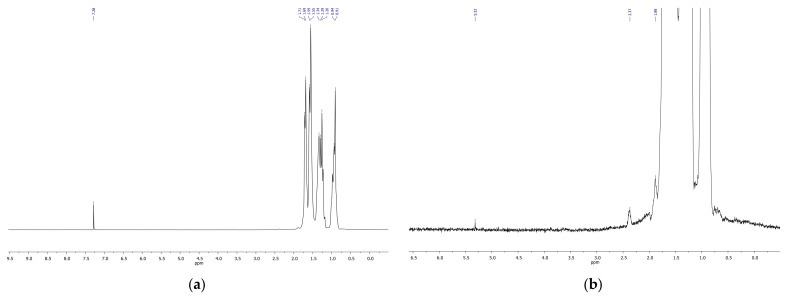
^1^H-NMR spectrum of the mixture of cis and trans-decalins after ozonation for 15 min in a wide range of chemical shifts (**a**) and in the narrow range of 0–6 ppm (**b**).

**Figure 9:**
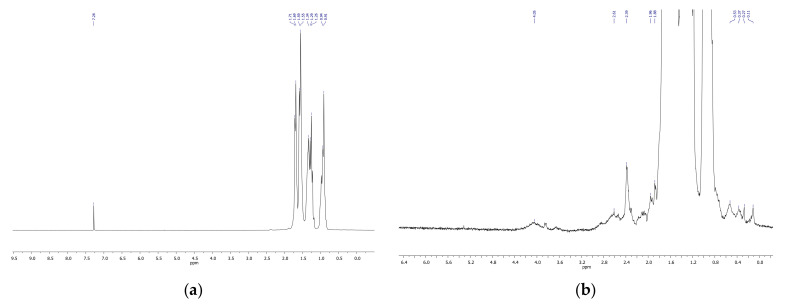
^1^H-NMR spectrum of the mixture of cis and trans-decalins after ozonation for 70 min in a wide range of chemical shifts (**a**) and in the narrow range of 0–6 ppm (**b**).

NMR spectroscopy confirmed the results obtained by IR spectroscopy. NMR spectra demonstrated the formation of diverse oxygenated products in the course of decalin ozonation, including isomers of hydroxylated products, ketones, hydroxyketones, and carboxylic acids (Figure 7, Figure 8 and Figure 9). The spectra of the initial isomer mixture (Figure 7) exhibits chemical shifts corresponding to cis and tans-decalin isomers [21,22]. Treatment of decalin with ozone for 15 min led to a low-intensity peak at 2.37 ppm related to protons in CH_2_COOH (acidic) and CH_2_CHO (aldehyde) groups (Figure 8).

Ozonation for 70 min resulted in an increase of the intensity of the peak at 2.37 ppm and to the appearance of other peaks from 2 to 3 ppm, i.e., protons at CH_2_-groups connected to COOH fragments (Figure 7). The lines at 4 ppm (Figure 9) provide evidence for the formation of alcohols containing CH_2_OH groups.

Titration of the water extract showed that 1 L of decalin after ozonation for 19 min contained about 8 mmoles of water-soluble acids. If we take into account that only 5% of the products was extracted, then the amount of insoluble (in water) products for the ozonation time of 19 min should be 0.16 mol/L, whereas for the ozonation time of 70 min, the concentrations of soluble and insoluble products should increase to 0.03 and 0.7 mol/L, respectively.

Earlier ozonation of decalin isomers has been studied [16]. The key stage of the process was assumed to be the activation of secondary carbon atoms by converting decalin into carbonyl compounds. Hydroxydecalin and the corresponding ketone were identified as the major products. Hydrogen transfer as the first step in the hydroxylation process was also considered [17].

The data obtained in this work provides grounds to postulate the mechanism of decalin ozonation presented as follows (RH^*^ stands for decalin) in agreement with schemes considered in the literature [23,24].

1. Cleavage of the mobile hydrogen atom H* in two successive acts:

RH^*^ + O_3_ → R^•^ + ^•^OH + O_2_ (1)

RH^*^ + ^•^OH → R^•^ + H_2_O (2)

2. Formation of a peroxide radical:

R^•^ + O_2_ → ROO^•^ (3)

Further transformations of R^•^ and ROO^•^ results in the formation of diverse products of the oxidative decomposition of decalin. The ozone in ozone-oxygen mixtures is a smaller part of the mixture compared to the oxygen present (in our case, the ozone content was equal to 100–150 mg per 1 L of the gas mixture, i.e., 4–6% of ozone). Given this fact, it can be assumed that ozone acts, most likely, as an activator of the oxidative process, which results in the formation of reactive organic radicals that involve oxygen in the chemical transformations of an organic compound.

Ozonolysis of alkenes occurs first, with the formation of two carbonyl groups via splitting of the C=C bond. [2,4]. The chemical properties of cycloalkanes resemble those of alkenes; therefore, the formation of carbonyl products at the initial stage of the ozonation process does not look strange. We believe, however, that two parallel mechanisms are possible in the case of decalin ozonolysis. The fast one results in the formation of carbonyl compounds, and the slow process leads to the formation of alcohols. Therefore, alcohols start to form at later stages of ozonation compared to carbonyl compounds. The formation of carboxy groups (acids) is possible as a result of disproportionation of peroxide radicals formed in reaction (3) by the reaction

X-CHR-O-O·+ X-CHR-O-O·→ X-CR = O + X-COOH + H_2_O (4)

where X and R denote carbon fragments of the decalin molecule.

We do not see any controversy in considering two mechanisms of decalin ozonation, via a secondary carbon atom or via a tertiary carbon atom, because both mechanisms seem to be realized in parallel. Indeed, taking into account the variety of products formed, including both products from ozonation of tertiary carbon atoms (9-hydroxydecalin isomers) and those obtained by oxidation of secondary carbon atoms (1-decalone, 2-decalone), both mechanisms may contribute to the reaction course. Unidentified carbonic acids can be produced as secondary products by both mechanisms.

## 3. Materials and Methods

Ozone was obtained by passing high-purity oxygen (99.99%, NII KM, Moscow, Russia) through a capillary discharge chamber (the chamber included 20 capillary tubes with an external diameter of 3 mm and a length of 50 cm, flow rate of oxygen 50 mL/s), which provided an ozone-oxygen mixture with an ozone concentration of up to ~160 mg/L. The composition of the ozone-oxygen mixture at the reactor inlet was controlled using a “Medozone 254/5” ozone concentration meter (Medozone, Moscow, Russia). Additionally, the composition of the ozone-oxygen mixture was determined spectrophotometrically with a Cary 50 spectrophotometer (Varian BV, Middelburg, Netherlands) using a cuvette (path length 0.5 cm) containing the ozone-oxygen mixture at the experiment temperature. The results obtained coincided with those measured with the analyzer, with an accuracy of ±2–5%. To obtain an ozone-oxygen gas mixture at a given concentration of ozone in the range from 15 to 100 mg/L, an automatic unit UOTA-60-01 “Medozone” (Medozone, Moscow, Russia) was used.

IR spectra of attenuated total reflectance (IR-ATR) were measured using a Nicolet Is50 Fourier transform spectrometer (ThermoFischer Scientific, Waltham, MA, USA) with an integrated ATR attachment. Conditions for measuring IR spectra were a resolution of 4 cm^−1^, with 32 scans. ^1^H-NMR spectra were measured with a Bruker AM-300 NMR spectrometer (Bruker GmbH, Karlsruhe, Germany). Measurement conditions for ^1^H-NMR spectra were a frequency of 300.13 MHz using CDCl_3_ as the solvent.

Decalin saturation with ozone was performed by bubbling the ozone-oxygen mixture at a rate of 2 mL/s, while periodically determining the ozone content in the solution from its UV-visible absorption spectrum. The products were extracted with 1 mL of water from decalin (1 mL) at room temperature under stirring, with an extraction time of 10 min.

Optical spectra were recorded using a Cary 50 spectrophotometer. To register the spectrum, a portion of the ozonated solution was transferred to a quartz cuvette with a tightly fitting plug. Possible ozone losses during the time interval, during which three samples were taken, did not exceed 3%. All measurements were made at room temperature.

## 4. Conclusions

In comparison to alkanes, the cyclic structure and the presence of a mobile hydrogen atom at the tertiary carbon atom and secondary carbon atoms in decalin dramatically changed the ability of this saturated hydrocarbon to be oxidized by ozone. Ozonation of decalin may be considered a model of the oxidation of a cyclic saturated molecule mimicking the polycyclic compounds present in heavy oil fractions such as asphaltenes. Therefore, one may anticipate that the behaviour of asphaltenes and heavy residues under ozonation may be similar to the oxidation of decalin by ozone. Thus, we are planning to present a separate study of the reactions occurring with asphaltenes and heavy residues under the action of ozone.

## Figures and Tables

**Table 1:** Assignment of absorption bands in IR spectra of cis and trans-decalins, initial decalin (equilibrium mixture of cis and trans-isomers) and decalin after ozonation for 15 and 70 min.

Entry	Vibration Frequencies, cm^−1^		
	Initial Decalin (Equilibrium Mixture of Cis- and Trans-Isomers) *	Decalin After Ozonation for 15 min	Decalin After Ozonation for 70 min
1	-	3450, 3250 (O-H stretching)	Broad band 3600-2900 (O-H stretching)
2	2915 (C-H stretching)	2915	2915
3	2848 (C-H stretching)	2848	2849
4	2660 (C-H stretching)	2660	2660
5	-	1717 (C=O stretching)	1716 (C=O stretching)
6	1458 (C-H in plane bending)	1458	1458
7	1447 (C-H in plane bending)	1447	1447
8	1374 (C-H in plane bending)	1374	1374
9	1346 (C-H in plane bending)	1346	1346
10	1332 (C-H in plane bending)	1333	1333
11	1305 (C-H in plane bending)	1306	1306
12	1252 (C-H in plane bending)	1252	1252
13	1224 (C-H in plane bending)	1224	1224
14	1168 (C-H in plane bending, C-C stretching)	1168	1168
15	1154 (C-H in plane bending, C-C stretching)	1154	1154
16	1138 (C-H in plane bending, C-C stretching)	1138	1138
17	1071 (C-C stretching)	1071	1071
18	1056 (C-C stretching)	1056	1056
19	1028 (CCC trigonal bending)	1028	1028
20	1010 (CCC trigonal bending)	1010	1010
21	-	-	988, 950 (C-O stretching)
22	971 (C-H out of plane bending)	971	971
23	924 (C-H out of plane bending)	924	924
24	873 (C-H out of plane bending)	873	873
25	853 (CCC ring breathing, C-H out of plane bending)	853	853
26	839 (C-H out of plane bending)	839	839
27	824 (C-H out of plane bending)	824	825
28	800 (C-H out of plane bending)	800	800
29	599 (CCC in plane bending)	599	599
30	526 (CCC in plane bending)	526	552, 526
31	511 (CCC in plane bending)	511	512
32	491	-	-
30	477	476	
33	465	466	
34	455 (CCC in plane bending)		
35	445 (CCC in plane bending)	442	437
36	427	-	-
37	419 (CCC in plane bending)	-	418

* National Institute of Advanced Industrial Science and Technology of Japan (AIST), Spectral Database for Organic Compounds (SDBS).

## Data Availability

Not applicable.

## Supplementary Materials

The following are available online. The supporting information file includes figures with transmission IR spectra and ^1^H-NMR spectra of decalin before and after ozonolysis: Figure S1. Transmission IR spectra of the initial decalin (1) and decalin after ozonation for 70 min (2). Figure S2. IR spectra in the region of C=O stretching vibrations for decalin ozonated for 19 min before (1) and after extraction with water (2). Figure S3. The mass-spectrum of the mixture formed after 70 min of ozonation of decalin. Table S1. Comparison and assignment of absorption bands in IR spectra of cis- and trans-decalins, initial decalin (equilibrium mixture of cis- and trans-isomers) and decalin after ozonation for 15 and 70 min.
