# Supplementary material for: Ozonation of Decalin as a Model Saturated Cyclic Molecule: A Spectroscopic Study
Source: Molecules. 2021 Sep 13;26(18):5565. doi: 10.3390/molecules26185565 (PMC8469405; doi:10.3390/molecules26185565)
Supplement: Supplementary file 1 [file molecules-26-05565-s001.zip › molecules-1349698-supplementary.pdf]

**Supporting Information**

**Ozonation of decalin as a model saturated cyclic molecule**

Boris G. Ershov, Nadezhda M. Panich, Gennadii L. Bykov, Alexander L. Kustov[2,3,4], Vladimir Krasovsky, Leonid M. Kustov

[1] A.N. Frumkin Institute of Physical Chemistry and Electrochemistry RAS, 119071, 31 Leninsky Prospect, bldg. 4, Moscow, Russia

[2] N.D. Zelinsky Institute of Organic Chemistry RAS, 47 Leninsky prosp., Moscow 119991 Russia

[3] National University of Science and Technology MISiS, 4 Leninsky prosp., Moscow 119049, Russia

[4] Chemistry Department, Moscow State University, 1 Leninskie Gory, bldg. 3, Moscow, 119991 Russia

***IR-spectroscopy data***

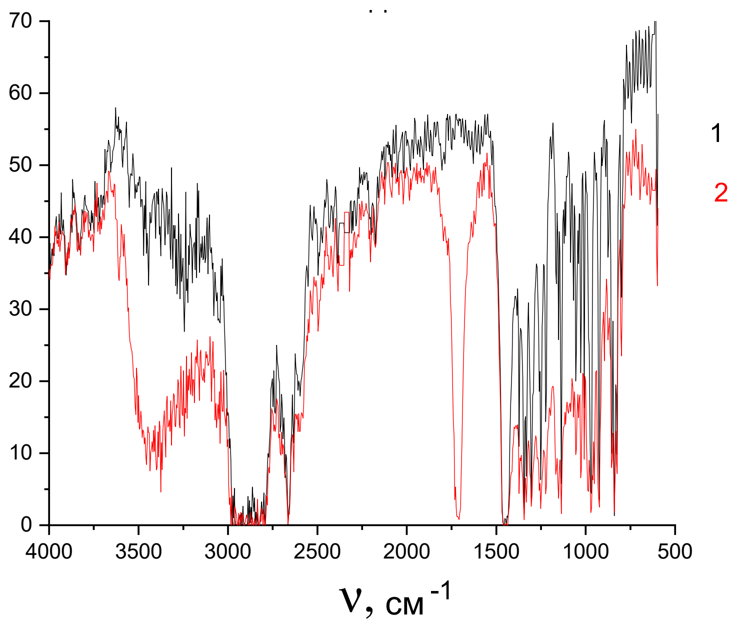


Figure S1. Transmission IR spectra of the initial decalin (1) and decalin after ozonation for 70 min (2).

**Table S1.** Comparison and assignment of absorption bands in IR spectra of cis- and trans-decalins, initial decalin (equilibrium mixture of cis- and trans-isomers) and decalin after ozonation for 15 and 70 min. *National Institute of Advanced Industrial Science and Technology of Japan (AIST), Spectral Database for Organic Compounds (SDBS).

| Entry | Vibration frequencies, cm-1 | | | | |
|---|---|---|---|---|---|
| | Initial decalin (equilibrium mixture of cis- and trans-isomers) | cis-Decalin [19, 21] | Trans-Decalin [20, 21] | Decalin after ozonation for 15 min | Decalin after ozonation for 70 min |
| 1 | - | - | - | 3450, 3250 (O-H stretching) | Broad band 3600-2900 (O-H stretching) |
| 2 | 2915 | 2975, 2912 (C-H stretching) 2924 | 3056, 2913, 2900 (C-H stretching) 2919 | 2915 | 2915 |
| 3 | 2848 | 2838, 2781, 2770, 2750, 2731, 2707 (C-H stretching) 2861, 2736, 2709 | 2875, 2838, 2794, 2750, 2713, 2700 (C-H stretching) 2852, 2793, 2707 | 2848 | 2849 |
| 4 | 2660 | 2675, 2663, 2644 (C-H stretching) 2680, 2655, 2646 | 2681, 2663, 2631 (C-H stretching) 2581 (1350+1219) 2694, 2678, 2660, 2632, 2598, 2580 | 2660 | 2660 |
| 5 | - | - | - | 1717 (C=O stretching) | 1716 (C=O stretching) |
| 6 | 1458 | 1463 (C-H in plane bending) 1466, 1460, 1452 | 1463 (C-H in plane bending) 1459 | 1458 | 1458 |
| 7 | 1447 | 1437 (C-H in plane bending) 1447 | 1450, 1413 (C-H in plane bending) 1449 | 1447 | 1447 |
| 8 | 1374 | 1381 (C-H in plane bending) 1374 | 1388 (C-H in plane bending) | 1374 | 1374 |
| 9 | 1346 | 1350 (C-H in plane bending) 1345 | 1350 (C-H in plane bending) 1347 | 1346 | 1346 |
| 10 | 1332 | 1319 (C-H in plane bending) 1336, 1316 | 1325(C-H in plane bending) 1333 | 1333 | 1333 |
| 11 | 1305 | 1305 (C-H in plane bending) 1303 | 1300 (C-H in plane bending) 1307 | 1306 | 1306 |
| 12 | 1252 | 1263 (C-H in plane bending) 1295, 1261 | 1288, 1250 (C-H in plane bending) 1253 | 1252 | 1252 |
| 13 | 1224 | 1219 (C-H in plane bending, C-C stretching) | 1225 (C-H in plane bending) 1225 | 1224 | 1224 |

| | | | | | |
|---|---|---|---|---|---|
| 14 | 1168 | 1175 (C-H in plane bending, C-C stretching) 1168 | - | 1168 | 1168 |
| 15 | 1154 | 1150 (C-H in plane bending, C-C stretching) | 1150 (C-H in plane bending, C-C stretching) 1154 | 1154 | 1154 |
| 16 | 1138 | 1114 (C-C stretching) | 1138(C-H in plane bending, C-C stretching) 1139 | 1138 | 1138 |
| 17 | 1071 | 1088. 1075 (C-C stretching) 1071 | - | 1071 | 1071 |
| 18 | 1056 | 1050 (C-C stretching) | 1063 (C-C stretching) 1057 | 1056 | 1056 |
| 19 | 1028 | - | 1025 (CCC trigonal bending) 1029 | 1028 | 1028 |
| 20 | 1010 | 1012 (CCC trigonal bending) 1011 | - | 1010 | 1010 |
| 21 | - | - | - | - | 988, 950 (C-O stretching) |
| 22 | 971 | 981, 963 (C-H out of plane bending) 979, 969 | 990, 975 (C-H out of plane bending) 988, 972 | 971 | 971 |
| 23 | 924 | 938 (C-H out of plane bending) | 925 (C-H out of plane bending) 925 | 924 | 924 |
| 24 | 873 | 895, 881 (C-H out of plane bending) 874 | - | 873 | 873 |
| 25 | 853 | 850 (CCC ring breathing) 854 | 863 (C-H out of plane bending) | 853 | 853 |
| 26 | 839 | 835 | 838(C-H out of plane bending) 840 | 839 | 839 |
| 27 | 824 | 830 (C-H out of plane bending) | 825 (C-H out of plane bending) 825 | 824 | 825 |
| 28 | 800 | 800, 743 (C-H out of plane bending) | 800 (C-H out of plane bending) 804 | 800 | 800 |

| | | | | | |
|---|---|---|---|---|---|
| | | 800, 793 | | | |
| 29 | 599 | 600 (CCC in plane bending) 600 | - | 599 | 599 |
| 30 | 526 | - | 525 (CCC in plane bending) 527 | 526 | 552, 526 |
| 31 | 511 | 512 (CCC in plane bending) | - | 511 | 512 |
| 32 | 491 | | - 481 | - | - |
| 30 | 477 | | - | 476 | |
| 33 | 465 | | - | 466 | |
| 34 | 455 | | 450 (CCC in plane bending) | | |
| 35 | 445 | 444 (CCC in plane bending) | - | 442 | 437 |
| 36 | 427 | - | - | - | - |
| 37 | 419 | - | 400 (CCC in plane bending) | - | 418 |

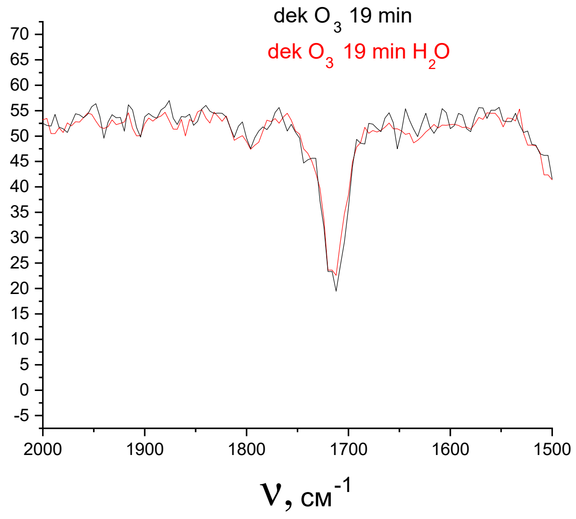


Figure S2. IR spectra in the region of C=O stretching vibrations for decalin ozonated for 19 min before (1) and after extraction with water (2).

### *Mass-spectrometry data for the mixture obtained after 70 min of ozonation*

Figure S3 presents the mass-spectrum of the mixture formed after 70 min of ozonation of decalin.

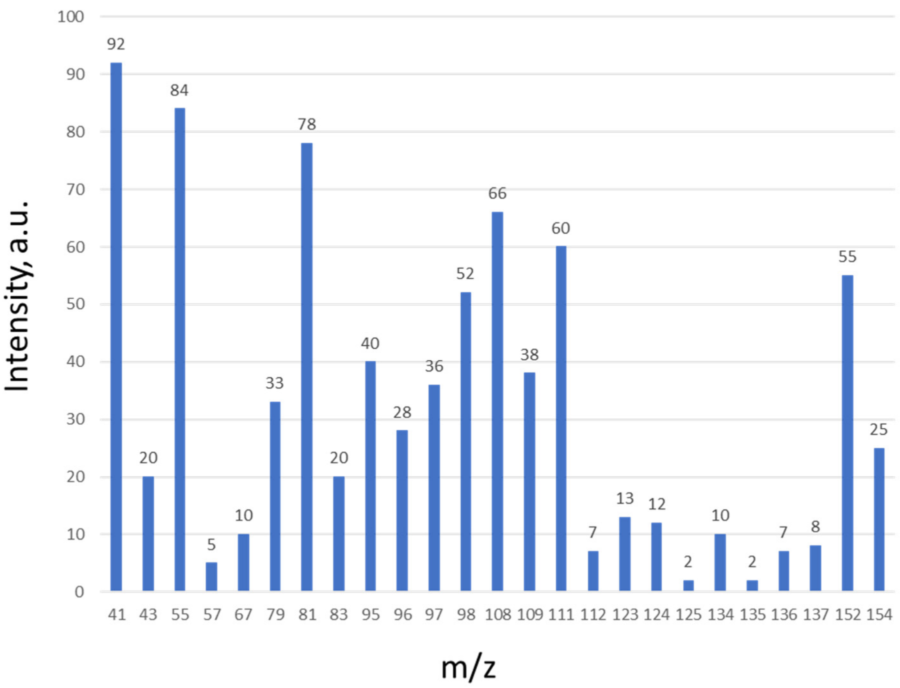


Figure S3. The mass-spectrum of the mixture formed after 70 min of ozonation of decalin.

The following major products can be identified by mass-spectrometry (70 eV) while changing the time of ozonation, although the overall number of products reaches at least 15-16:

Decalin after ozonation for 70 min

m/z: 154 (25), 152 (55), 137 (8), 136 (7), 135 (2), 134 (10), 125 (2), 124 (12), 123 (13), 112 (7), 111 (60), 110 (33), 109 (38), 108 (66), 98 (52), 97 (36), 96 (28), 95 (40), 83 (20), 81 (78), 79 (33), 77 (6), 70 (12), 69 (10), 67 (67), 57 (5), 56 (8), 55 (84), 43 (20), 41 (92).

2-Decalone

m/z: 152 (60, M'), 137 (9), 136 (6), 135 (2), 134 (12), 123 (15), 110 (43), 109 (28), 108 (96), 97 (26), 96 (38), 95 (50), 81 (98), 79 (43), 67 (88), 55 (96), 41 (100)

1-Decalone

m/z: 152 (51, M'), 137 (5) , 136 (9), 134 (16), 124 (13), 123 (26), 111 (10), 110 (63), 109 (100), 108 (28), 97 (40), 95 (20), 81 (85), 67 (95), 55 (52), 41 (80).

(E)-9-Decalol

m/z: 154 (26, M'), 136 (2), 125 (2), 112 (5), 111 (100), 98 (50), 97 (25), 83 (19), 70 (11) , 67 (15), 57 (5), 56 (9), 55 (47), 43 (20).

(Z)-9-Decalol

m/z: 154 (23, M'), 136 (2), 125 (2), 112 (9), 111 (100), 98 (53), 97 (27), 95 (3), 83 (22), 81 (6), 79 (9), 77 (7), 70 (14), 69 (12), 67 (17), 55 (53), 43 (23).
